# EDUCATIONAL CHALLENGES OF CHILDREN IN SKIPPED GENERATION HOUSEHOLDS IN NIGERIA

**DOI:** 10.1093/geroni/igz038.2458

**Published:** 2019-11-08

**Authors:** Bayode I Popoola, Funmi Togonu-Bickersteth, Joshua O Aransiola, Akinjide Akintomide, Opeyemi Ekundayo

**Affiliations:** 1 OBAFEMI AWOLOWO UNIVERSITY, ILE IFE, OSUN, Nigeria; 2 Obafemi Awolowo University, Ile-Ife, Ile-Ife, Osun State, Nigeria

## Abstract

The paper examined the challenges of accessing education by Nigerian children raised in an unusual family context, the skipped generation households. Specifically, it determined the proportion of Nigerian school-age children in skipped generation households enrolled in the formal school system and investigated the children’s perception of the effect of living in skipped generation household on their education. The paper also ascertained regional differences in education challenges experienced by children in skipped generation households. The study adopted the descriptive survey design. The research procedure involved the collection of primary data through the administration of a survey questionnaire on 2144 indexed children from three purposively selected states representing the three major ethnic groups in Nigeria. The results showed that 88.2% of Nigerian children in skipped generation households were enrolled in the formal school system, and that significant regional variations existed in school attendance by children in skipped generation households. Also, a substantial majority (74.1%) of grandchildren reported that living in SGHs had no negative effect on their academic performance. The specific education challenges of school-going children in skipped generation households included having to do assignment alone, not getting enough time to study, and difficulty in paying school fee. The results further indicated that the education challenges experienced by children in skipped generation households were significantly different across the selected states which constitute the study area. The paper highlighted the need for government to improve the welfare of older adults in Nigeria, especially those who serve as custodial grandparents for their grandchildren.

